# The effects of mask applied aromatherapy on allergic rhinitis symptoms, fatigue, and quality of life related to allergic rhinitis in the COVID-19 era: a randomized controlled trial

**DOI:** 10.7586/jkbns.24.021

**Published:** 2024-08-22

**Authors:** Jihoo Her, Myung-Haeng Hur

**Affiliations:** 1 Gimcheon University, Gimcheon, Korea; 2 College of Nursing, Eulji University, Uijeongbu, Korea

**Keywords:** Rhinitis, allergic, Aromatherapy, Eucalyptus oil, Masks, Quality of life

## Abstract

**Purpose:**

Even after allergic rhinitis is diagnosed and treated, various symptoms such as runny nose, sneezing, and itchy nose recur periodically due to various environmental factors.

**Methods:**

This randomized controlled trial was conducted to confirm the effectiveness of mask-based aromatherapy as a method of alleviating allergic rhinitis during the coronavirus disease 2019 (COVID-19) pandemic. The study participants were adults between the ages of 19 and 60 who were diagnosed with allergic rhinitis and had a Total Nasal Symptom Score (TNSS) of 2 or more. Participants were randomly assigned to an aromatherapy group that was provided aromatherapy essential oils and a control group that did not receive aromatherapy. Participants in the aromatherapy group were asked to drop an aromatherapy essential oil onto a disposable face mask and wear it twice a day for two weeks.

**Results:**

The aromatherapy group experience significant alleviation of allergic rhinitis symptoms (visual analogue scale, F = 11.22, *p* < .001; TNSS, F = 15.82, *p* < .001). The aromatherapy group also showed significantly higher improvements in fatigue (F = 8.28, *p* < .001), allergic rhinitis-specific quality of life (F = 11.12, *p* < .001).

**Conclusion:**

The oils used in this study appeared to be effective in relieving the symptoms of allergic rhinitis. In particular, the mask drop inhalation method was applied in light of mandatory mask-wearing due to the global COVID-19 pandemic during this study period. Mask drop inhalation is a method of inhaling oil close to the nose and is considered an effective method for reducing the inconvenience of applying oil.

## INTRODUCTION

It has been reported that approximately 339 million people worldwide suffer from allergic rhinitis (AR), which is known as one of the top 20 diseases causing inconvenience throughout one's life [1,2]. AR is known to affect 15%-20% of adults [1-3]. The symptoms of AR include an itchy nose, repeated sneezing, a runny nose, and a stuffy nose [4,5].

AR symptoms can persist for an extended duration and may lead to headaches, sleep disturbances, chronic fatigue, and ultimately a diminished quality of life (QoL) [6-8]. Consequently, various complementary alternative medicine (CAM) has been tried to alleviate AR symptoms [9,10]. These CAM therapies have been commonly used by 30%-60% of AR patients in various countries, including the United States [9,11,12]. This indicates that patients are satisfied with the effectiveness of CAM for AR [13,14]. Aromatherapy is a commonly used CAM for AR [15]. Essential oil extracted from plants stimulates nerve pathways from the olfactory epithelium to the limbic system, contributing to its healing effects by balancing the autonomic nervous system [16,17].

Meanwhile, as the use of face masks increases due to the coronavirus disease 2019 (COVID-19) pandemic, research into applying aromatherapy to face masks has been tried. The aromatherapy applied to the mask was reported to have reduced stress and anxiety [18-20], and also alleviated breathlessness caused by wearing the mask, resulting in a significant improvement in the comfort of breathing [20]. Using a face mask reduces the sensitization to antigens, which are the cause of AR symptoms [21-23]. However, there are still reports of subjects whose AR symptoms have not decreased despite the use of masks [24]. Therefore, there is a need for methods to alleviate AR symptoms and improve QoL by applying CAM such as aromatherapy, which can be additionally used on masks to more effectively control AR. Therefore, we aimed to confirm the effects of aromatherapy applied to masks on AR symptoms and AR-related QoL (ARSQOL).

The purpose of this study is to investigate the effects of aromatherapy dropped onto masks on AR symptoms, fatigue, and rhinitis-related QoL in adults with AR. The specific objectives are as follows:

1) To investigate the effects of aromatherapy dropped onto masks on AR symptoms.

2) To investigate the effects of aromatherapy dropped onto masks on fatigue.

3) To investigate the effects of aromatherapy dropped onto masks on rhinitis-related QoL.

## METHODS

### 1. Study design

**Figure 1.**
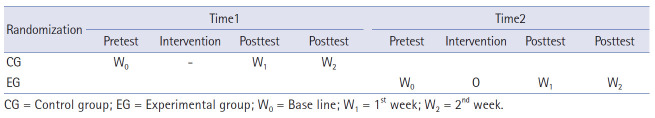
Study design.

We conducted a randomized controlled trial of convenience sample of AR patient to confirm the effectiveness of mask drop aromatherapy as a method of alleviating AR during the time when coronavirus infection was prevalent. The experiments in this study adopted a non-synchronized design with two groups. The research design is shown in Figure 1.

### 2. Participants

The study participants were adults between the ages of 20 and 60 who were diagnosed with AR and had a Total Nasal Symptom Score (TNSS) of 2 or more. To recruit participants, a recruitment notices were posted on the bulletin board in the Seoul National University Bundang Hospital and the intranet of Hyundai Motor Namyang Research Center. from June 10 to 30, 2020. All participants voluntarily expressed their intention to participate.

We calculated the sample size using G-power 3.1.9.7 [25], based on a F test, repeated measures ANOVA, within-between interaction with a significance level of 0.05, an effect size of 0.259, a statistical power of 0.95, number of groups of 2, number of measurements 3, correlation among repeated measures 0.3 and nonsphericity eta ε 1. And then total sample size was 54. The sample size required each group to have 30 participants while considering about 10% dropout rate. Finally, participants were randomly allocated to the aroma group (n = 30) and control group (n = 30) using Excel's random number generation method. No information regarding group assignment was provided to the participants.

The participants inclusion criteria were adults aged between 19 and 60 years, with no abnormality in the sense of smell and with a TNSS of 2 or more. Exclusion criteria were those who are sensitive or reluctant to aroma essential oil, who are currently pregnant, and with diseases, such as hypotensive, heart, liver, or kidney disease.

### 3. Experimental setup

#### 1) Selection of essential oils for mask drop aromatherapy

The selection of the aromatherapy essential oils used in the experimental treatment was based on the recommendation of an international aromatherapist, following established guidelines [26]. In this study, an aromatherapy essential oil was blended with eucalyptus, lemon, Roman chamomile, and peppermint in a ratio of 2:2:1:0.5. For the safety of the participants, we carefully considered factors such as the product's extraction area and method, herb expiration date, and more when selecting the oil. As a result, we chose a product from Neumond (website: http://neumond.de/) and purchased it through Geunalae in Korea (website: https://www.geunalae.co.kr).

#### 2) Experimental intervention

Participants in the aroma group were asked to drop aromatherapy essential oil onto a disposable face mask and wear it twice a day for two weeks. Considering the social situation mandating the use of masks due to COVID-19, one drop of aroma oil was applied to disposable masks in the morning and afternoon. Additionally, at the bedtime, one drop of oil was added to the pillows for the aroma group. The participants were instructed to wear masks even when they were at home. The basis for our intervention was founded on literature review guidelines that recommend a 2-3 week intervention for aromatherapy in individuals with health problems [16,26]. Additionally, for the dry inhalation method, it is suggested to inhale 1-5 times as needed, or to use continuous inhalation methods such as an aroma necklace [27]. Based on these guidelines and after consulting with a professional aromatherapist, we applied the oil to the mask twice a day and once before sleep.

The aroma group was provided with a pre-prepared, pre-packaged bag containing a questionnaire, disposable face mask, oil, and instructions for use. The aroma group was educated on how to use the essential oils, precautions, storage methods, and potential side effects. They were also instructed to immediately stop using the oils and contact the investigator if side effects occurred, such as headache or skin irritation.

### 4. Outcomes

The primary outcome of this study was AR symptoms and the secondary outcomes were fatigue and AR-specific QoL.

#### 1) AR symptoms

Patient's subjective symptoms for AR can be evaluated using validated patient-reported outcome measures [28] which commonly include the TNSS and Visual Analogue Scale (VAS) [29]. Therefore, we measured AR symptoms using the VAS and TNSS [30]. The VAS represents the overall symptoms as perceived by the individual on a scale of 0 to 10, while the TNSS scores AR symptoms across different categories. The VAS is expressed on a vertical line of 10 cm, ranging from no discomfort (0) to very severe (10). The TNSS includes nasal itching, sneezing, watery runny nose, and stuffy nose. These symptoms are graded on a 4-point scale from 0 to 3 respectively: 0 (nothing at all), 1 (mild), 2 (moderate), and 3 (severe). At the time of development, the reliability of the TNSS was Cronbach's ⍺ = .87. In this study, the reliability of the TNSS was Cronbach's ⍺ = .69.

#### 2) Fatigue

In this study, fatigue was assessed using a tool [31], which was a modified version of the questionnaire standardized by the Industrial Fatigue Research Committee of the Japan Industrial Hygiene Association (1988). The fatigue self-awareness survey consisted of 30 questions, each rated on a 4-point Likert scale ranging from 1 (not at all) to 4 (always). At the time of development, the reliability of this tool was Cronbach's α = .82, and the reliability in study [31] was Cronbach's α = .89. In this study, the reliability of the tool was Cronbach's ⍺ = .98.

#### 3) ARSQOL

The ARSQOL questionnaire [32] was used in this study with the authors' consent to evaluate the QoL of adults with AR. The questionnaire assesses five categories of discomfort caused by symptoms of AR, including physical, mental, social function, daily life, and other life areas subjectively perceived by the patient. The ARSQOL tool includes 26 questions on a 5-point Likert scale. At the time of development, Cronbach's α value for all items = .95. In this study, the reliability of the tool was Cronbach's ⍺ = .96.

### 5. Data collection

To alleviate the potential threat of internal validity due to experimental contamination among individuals working in the same institution, the study was conducted with a time lag. Intervention and data collection were conducted first for the control group, and followed by the experimental group. In the control group, measurements were taken 2 times at 1-week intervals without any treatment.

To ensure the smooth progress of the study, participants were instructed to set an alarm on their mobile phones at the specified time for adapting to the aroma oil. Additionally, participants were instructed to contact the researcher if they were unable to apply the aroma oil at the specified time or encountered such a situation. After the intervention, the participants were sent a text message asking them to complete the questionnaires 1^st^ and 2^nd^ week after intervention. Once they finished filling out the questionnaire, they sent a text message to the researcher. The participants were then instructed to place the completed questionnaires in the provided brown envelopes within 1-2 days. The researcher personally collected these envelopes at the designated location. The collection process occurred twice, after the Week 1 and Week 2 surveys, respectively.

### 6. Statistical analysis

In this study, the data were managed using the Excel program, and the analysis was performed using SPSS 26.0 (IBM Corp., Armonk, NY, USA) statistical software. The significance level (α) was set at 0.05. Measurements were expressed as mean ± standard deviation, and numerical values, and percentages. Homogeneity tests were conducted using the χ^2^-test and t-test. The effects of pre- and post-treatment on AR symptoms, fatigue, and ARSQOL in the two groups were analysed using the t-test and repeated measures ANOVA. The Bonferroni post-hoc method was performed to analyse the time-point differences.

If Mauchly's sphericity test was met, within-subject tests were conducted; otherwise, multivariate tests using Wilks' Lambda were performed. The reliability of the measurement tools was assessed using Cronbach's α coefficient.

### 7. Ethical considerations

This study was approved by the Institutional Review Board of the Eulji University (Approval no. EUN20-014-01) and registered with the Clinical Research Information Service (KCT0006323). The study objectives and procedures were explained to all participants, and informed consent was obtained from each of them before the study began. The collected data was kept confidential and managed using a unique identification number assigned to each participant, and was used only for the purposes of this study.

## RESULTS

### 1. Homogeneity test of general characteristics of participants

**Figure 2.**
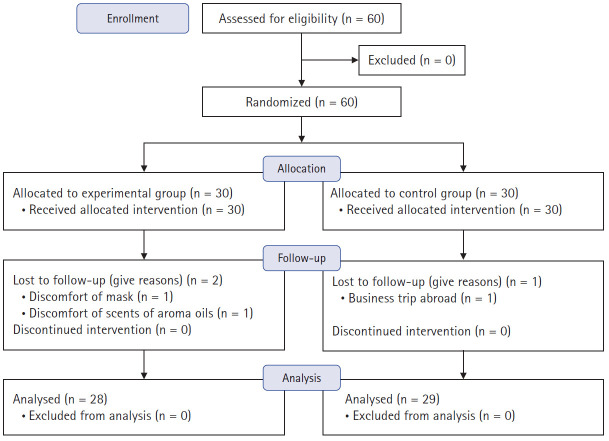
Flow diagram.

The study flow diagram displays assignment with 30 participants to each two groups; and 28 in the aroma group, and 29 in the control group after dropouts (Figure 2). There were no significant differences among the two groups in general characteristics and baseline of subjective rhinitis symptoms, TNSS, fatigue, and ARSQOL; therefore, the two groups were considered to be homogeneous (Table 1).

### 2. Primary outcomes

#### AR symptoms

There were no significant differences observed in AR scores (VAS, TNSS) between the aroma group and the control group at baseline. After experimental treatments, the AR scores (VAS, TNSS) of the aroma group were significantly higher than those of the control group at the 1^st^ and 2^nd^ week (VAS: t = 2.38, *p* = .021; t = 3.88, *p* < .001, respectively). (TNSS: t = 3.05, *p* = .004; t = 3.91, *p* < .001, respectively). We observed a significant difference in the group-by-time interaction effect (VAS: F = 11.22, *p* < .001, TNSS: F = 15.82, *p* < .001) (Table 2).

### 3. Secondary outcomes

#### 1) Fatigue

There were no significant differences observed in fatigue scores between two groups at baseline(W0) and 1^st^ , 2^nd^ week (W_1_, W_2_) after intervention (W_0_: t = −1.14 , *p* = .259, W_1_: t = .72, *p* = .473, W_2_: t = .92, *p* = .364). But we observed a significant difference in the group-by-time interaction effect (F = 8.28, *p* < .001) (Table 2).

#### 2) ARSQOL

There were no significant differences observed in ARSQOL scores between two groups at baseline (W_0_) and 1^st^, 2^nd^ week (W_1_, W_2_) after intervention (W_0_: t = 1.25, *p* = .217, W_1_ : t = −1.15, *p* = .255, W_2_: t = −1.86, *p* = .068). But we observed a significant difference in the group-by-time interaction effect (F = 11.12, *p* < .001) (Table 2).

## DISCUSSION

We found that AR symptoms were significantly lower in the aroma group compared to the control groups. Additionally, the ARSQOL score was higher in the aroma group compared to control group. In this study, the aromatherapy blend includes Eucalyptus, Roman Chamomile, Lemon, and Peppermint. Each oil contains specific chemical constituents and possesses various mechanisms for relieving respiratory symptoms. Firstly, Eucalyptus oil predominantly contains 1,8-cineole (69.1%), which exhibits antibacterial and anti-inflammatory effects [16,26]. Secondly, Roman Chamomile oil consists of 1,8-cineole (0.5%-25.0%) and possesses anti-inflammatory properties [16,26]. Thirdly, Lemon oil, with its component limonene (62.1%-74.5%), demonstrates antiseptic and antimicrobial effects [16,26]. Lastly, Peppermint oil is composed of menthol (40.0%), which has antiseptic and antispasmodic properties [26].

Our results were consistent with previous studies that applied aromatherapy to AR using traditional inhalation methods [33-35]. In these studies, the method of intermittently inhaling aroma oil was used, but this can be inconvenient because it requires putting on and taking off a mask. Our study alleviated this inconvenience by applying the aroma oil directly to the mask.

Recently, it has been reported that the increasing use of masks can reduce exposure to antigens, which are the cause of AR [21-23]. However, there are still people who experience AR symptoms even while wearing a mask. Additionally, due to the discomfort and stuffiness of masks, more people are choosing not to use them [36]. Therefore, a method of applying aromatherapy to masks is being attempted to increase comfort [37].

In this study, the experimental group that applied aroma oils to their masks showed a significant reduction in AR symptoms compared to the control group that only wore regular disposable masks. Therefore, it is considered that applying aroma oil to masks will be more effective in alleviating AR symptoms and improving their QoL.

Persistent symptoms of AR may cause mood changes, such as depression, anxiety, and work or academic disorders [38,39], which can significantly impact an individual's QoL [8]. Therefore, the significant improvement in the QoL seen in our study may be attributed to the decrease in the discomfort caused by rhinitis symptoms. Furthermore, considering the results of previous studies where applying aromatherapy to masks improved breathlessness and increased QoL [20], our study's findings can be seen as consistent with this trend.

In this study, there were no cases of side effects. These findings suggest that aromatherapy dropped the masks may be an effective alternative therapy to help alleviate rhinitis symptoms, and improve QoL.

## CONCLUSION

In conclusion, a two-week aromatherapy may decrease AR symptoms and improve ARSQOL. This study also demonstrated that using masks can provide an easy and convenient method for applying aroma oils. Inhalation aromatherapy was shown to be an effective nursing intervention for alleviating AR symptoms and improving the QoL, especially in the context of the COVID-19 pandemic. However, further studies with a larger sample size and other essential oil therapies are needed to compare the findings of this study.

## Figures and Tables

**Table 1.** Homogeneity Testing of General Characteristics between Groups (N = 57)

Variables	Experimental group (n = 28)	Control group (n = 29)	χ^2^ or t	*p*
Age (yr)	35.30 ± 9.50	36.00 ± 8.41	-0.28	.784
Sex			0.49	.494
Male	17 (60.7)	15 (51.7)		
Female	11 (39.3)	14 (48.3)		
Residence type			0.04	.851
Apartment	19 (67.9)	19 (65.5)		
Others	9 (32.1)	10 (34.5)		
Smoking			1.15	.840
Yes	7 (25.0)	4 (13.8)		
No	21 (75.0)	25 (86.2)		
Symptom period (yr)	11.76 ± 8.31	13.31 ± 9.32	-0.66	.510
VAS	6.21 ± 2.08	6.28 ± 1.98	0.12	.909
TNSS	6.75 ± 2.40	6.90 ± 2.85	0.21	.835
Fatigue	59.21 ± 14.46	54.48 ± 16.76	-1.14	.259
ARSQOL	73.61 ± 19.26	80.52 ± 22.35	1.25	.217

Values are presented as the mean ± standard deviation or n (%). VAS = Visual analogue scale; TNSS = Total Nasal Symptom Score; ARSQOL = Allergic Rhinitis-Specific Quality of Life.

**Table 2.** Effects of Mask-based Aromatherapy on the Dependent Variables (N = 57)

Variables		Experimental group (n = 28)	Control group (n = 29)	t	*p*	F	*p*
VAS	W_0_	6.21 ± 2.08	6.28 ± 1.98	0.12	.909	Time 18.08	< .001
	W_1_	4.86 ± 1.98^†^	6.03 ± 1.76	2.38	.021	Group 5.76	.020
	W_2_	3.71 ± 2.51^†^	6.00 ± 1.91	3.88	< .001	G*T 11.22	< .001
TNSS	W_0_	6.75 ± 2.40	6.90 ± 2.85	0.21	.835	Time 34.49	< .001
	W_1_	4.46 ± 1.73^†^	6.21 ± 2.53*	3.05	.004	Group 6.29	.015
	W_2_	3.86 ± 2.27^†^	6.45 ± 2.71	3.91	< .001	G*T 15.82	< .001
Fatigue	W_0_	59.21 ± 14.46	54.48 ± 16.76	-1.14	.259	Time 9.94	< .001
	W_1_	54.21 ± 13.72^†^	57.59 ± 20.88	0.72	.473	Group 0.04	.834
	W_2_	49.82 ± 14.29^†^	53.83 ± 18.42	0.92	.364	G*T 8.28	< .001
ARSQOL	W_0_	73.61 ± 19.26	80.52 ± 22.35	1.25	.217	Time 32.85	< .001
	W_1_	90.57 ± 19.06^†^	84.14 ± 22.90	-1.15	.255	Group 0.369	.546
	W_2_	98.00 ± 19.54^†^	88.17 ± 20.24	-1.86	.068	G*T 11.12	< .001

Values are presented as the mean ± standard deviation. W_0_ = Baseline; W_1_ = 1^st^ week; W_2_ = 2^nd^ week; VAS = Visual analogue scale; TNSS = Total nasal symptom score; ARSQOL = Allergic rhinitis-specific quality of life.

† significant difference vs. W_0_ (*p* < .005, repeated measured ANOVA/ Bonferroni).

## Data Availability

The data that support the findings of this study are available from the corresponding author upon reasonable request.
